# Immortalization and Characterization of Porcine Macrophages That Had Been Transduced with Lentiviral Vectors Encoding the SV40 Large T Antigen and Porcine Telomerase Reverse Transcriptase

**DOI:** 10.3389/fvets.2017.00132

**Published:** 2017-08-21

**Authors:** Takato Takenouchi, Hiroshi Kitani, Shunichi Suzuki, Michiko Nakai, Dai-ichiro Fuchimoto, Mitsutoshi Tsukimoto, Hiroki Shinkai, Mitsuru Sato, Hirohide Uenishi

**Affiliations:** ^1^Division of Animal Sciences, Institute of Agrobiological Sciences, National Agriculture and Food Research Organization, Tsukuba, Japan; ^2^Faculty of Pharmaceutical Sciences, Tokyo University of Science, Noda, Japan

**Keywords:** porcine macrophages, immortalization, SV40 large T antigen, porcine telomerase reverse transcriptase, lentiviral vector

## Abstract

The domestic pig is an important agricultural animal, and thus, infectious diseases that affect pigs can cause severe economic losses in the global swine industry. Various porcine pathogens target macrophages, which are classical innate immune cells. Although macrophages basically protect the host from pathogens, they also seem to contribute to infectious processes. Therefore, cultured macrophages can be used to develop *in vitro* models for studying not only genes associated with porcine innate immunity but also the infectious processes of porcine pathogens. However, the availability of porcine macrophage cell lines is limited. In this study, we describe a novel immortalized porcine kidney-derived macrophage (IPKM) cell line, which was generated by transferring the SV40 large T antigen (SV40LT) and porcine telomerase reverse transcriptase (pTERT) genes into primary porcine kidney-derived macrophages using lentiviral vectors. The IPKM displayed a typical macrophage morphology and was routinely passaged (doubling time: about 4 days). These cells were immunostained for macrophage markers. In addition, they exhibited substantial phagocytosis of polystyrene microbeads and released inflammatory cytokines upon lipopolysaccharide (LPS) stimulation. Furthermore, the maturation and secretion of interleukin-1β were observed after nigericin-induced inflammasome activation in LPS-primed IPKM. These findings suggest that IPKM exhibit the typical inflammatory characteristics of macrophages. By transferring the SV40LT and pTERT genes using lentiviral vectors, we also successfully immortalized macrophages derived from the peripheral blood of a low-density lipoprotein receptor-deficient pig. These results suggest that the co-expression of SV40LT and pTERT is an effective way of immortalizing porcine macrophages.

## Introduction

Macrophages are classical innate immune cells that play a protective role in host defense against pathogens. They are myeloid lineage cells and circulate in the blood as monocytes before entering tissues and differentiating into macrophages with tissue- and niche-specific functions (1). Accumulating evidence has also revealed that tissue-resident macrophages include self-renewing embryo-derived populations (1). In addition to protecting the host, macrophages are a common target for various viral and bacterial pathogens and seem to contribute to the infectious processes of such pathogens (2). Therefore, *in vitro* systems involving cultured macrophages are useful models for studying pathogen infections and genes associated with innate immunity.

Since the domestic pig is an important agricultural animal, infectious diseases that affect swine, such as porcine reproductive and respiratory syndrome (PRRS), porcine epidemic diarrhea (PED), and bacterial infections, can cause severe economic losses in the global swine industry. The PRRS virus (PRRSV) is the causative agent of PRRS and targets porcine monocyte/macrophage lineage cells. CD163 is an essential receptor for this virus infection (3). The PED virus, a coronavirus, is the causative agent of PED and infects macrophages and disrupts their functions (4). In addition, bacterial pathogens, such as *Brucella* and *Salmonella*, also target macrophages in swine and impair their functions, which facilitate the invasion and proliferation of such pathogens (5, 6).

To promote virological and immunological studies in swine, several porcine macrophage cell lines have been developed (7–10). Among these, only three cell lines that were established by the transfection of primary porcine alveolar macrophages with the pSV3-neo plasmid, which carries genes for neomycin resistance and the SV40 large T antigen (SV40LT) (9), are commercially available from the American Type Culture Collection (ATCC Number: CRL-2843, CRL-2844, and CRL-2845). However, it is reported that these cell lines did not support the replication of PRRSV (11), suggesting that they do not possess all the features of primary alveolar macrophages. Thus, continuous efforts are being made to develop convenient porcine cell lines that retain the characteristics of primary macrophages.

In our previous study, we isolated macrophages from a mixed primary culture of porcine kidney tissue (12). Using these primary porcine kidney-derived macrophages (PKM), we attempted to produce an immortalized macrophage cell line in this study. As a result, we successfully generated a novel immortalized PKM (IPKM) cell line by transferring the SV40LT and porcine telomerase reverse transcriptase (pTERT) genes into PKM using lentiviral vectors. Our lentiviral transduction protocol might be a useful approach to the immortalization of primary porcine macrophages.

## Materials and Methods

### Ethics Statement

The protocols for the use of animals were approved by the animal care committee (#H28-P04) of the Institute of Agrobiological Sciences, National Agriculture and Food Research Organization (NARO). Low-density lipoprotein receptor knockout (LDLR-KO) pigs were conventionally reared at the Institute of Livestock and Grassland Science, NARO. The experiments involving lentiviral vectors were approved by the gene recombination experiment safety committee (#1036465) of the Institute of Agrobiological Sciences, NARO.

### Isolation of Primary PKM

In our previous study, the cells harvested from a mixed primary culture of porcine kidney tissue (from a 6-day-old crossbred female pig) were cryopreserved (12). The frozen porcine kidney cells were rapidly thawed at 37°C, split into three T-75 tissue culture flasks (Sumitomo Bakelite Co., Ltd., Tokyo, Japan), and cultured in growth medium composed of Dulbecco’s modified Eagle’s medium (Sigma, St. Louis, MO, USA) containing 10% heat-inactivated fetal bovine serum (Sanko Junyaku Co., Ltd., Tokyo, Japan), supplemented with 25 µM monothioglycerol (Wako Pure Chemical Industries, Ltd., Osaka, Japan), 10 µg/ml insulin (Sigma), 100 µg/ml streptomycin (Life Technologies, Carlsbad, CA, USA), 100 U/ml penicillin (Life Technologies), and 5 µg/ml Fungin (InvivoGen, San Diego, CA, USA). The culture medium was replaced every 3–4 days. After around 14 days, a sheet-like mixed cell monolayer formed, and macrophage-like cells began to actively proliferate on the cell sheet. The proliferating cells were loosely attached to the cell sheet and so were harvested from the culture supernatant by centrifugation (1,500 rpm for 5 min). Since the macrophages readily attached to non-tissue culture grade plastic dishes, they were selectively isolated from the other types of cells on the basis of this feature (13).

### Isolation of Primary Macrophages from the Peripheral Blood of a LDLR-KO Pig

The cells harvested from the mixed primary culture of porcine kidney tissue were passaged twice at a split ratio of 1:10, before being passaged once more at a split ratio of 1:6. No PKM were recovered from the supernatant of the resultant porcine kidney mixed culture. Thus, the macrophage-depleted-porcine kidney mixed culture, which mostly consists of epithelial and fibroblastic cells, was used as a source of feeder cells for the isolation of porcine blood-derived macrophages. Then, based on the findings of a previous study (14), 1.5 ml of heparinized peripheral blood that had been obtained from a 3-month-old male LDLR-KO pig (15) were directly added to the feeder cell culture in a T-75 flask, and the medium was replaced every 3–4 days. After around 10 days, blood-derived macrophage-like cells appeared among the feeder cells, actively proliferated, and were harvested from the culture supernatant by centrifugation (1,500 rpm for 5 min). The macrophages that became attached to non-tissue culture grade plastic dishes were used for the immortalization experiments.

### Immortalization of Primary Porcine Macrophages Using Lentiviral Vectors

Isolated primary porcine macrophages were cultured in 60-mm non-tissue culture grade plastic dishes (Sumitomo Bakelite Co., Ltd.). pLVSIN-EF1α neo vectors encoding SV40LT or pTERT were created by Takara Bio, Inc. (Shiga Japan). An artificial Kozak sequence was included before the start codon of each gene to ensure efficient translation (16). To generate lentiviruses, each of the pLVSIN vectors (2 µg) was co-transfected with packaging vectors (lentiviral high titer packaging mix) (3.25 µg) into Lenti-X 293T cells (Takara Bio, Inc.) (2 × 10^6^ cells/60-mm dish) using the TransIT-293 transfection reagent (Takara Bio, Inc.) according to the manufacturer’s instructions. Culture supernatants containing lentiviral particles were harvested on day 2, filtered through a membrane filter (pore size: 0.45 µm; Millipore Millex, Millipore Co., Billerica, MA, USA), and used for the porcine macrophage transduction procedures in the presence of 8 µg/ml of Polybrene (Sigma). After several weeks of culturing, proliferative cells appeared, which were then cultured with growth medium containing 800 µg/ml G418 (Thermo Fisher Scientific, Grand Island, NY, USA) to select resistant cells.

### Quantitative Real-time Reverse Transcription-Polymerase Chain Reaction (Q-PCR) Analyses

To detect SV40LT and pTERT mRNA expression, Q-PCR analyses were performed at Takara Bio, Inc., according to the company’s protocol.

### Immunocytochemistry

The macrophages were seeded in 8-well chamber slides (Asahi Glass Co., Ltd., Tokyo, Japan) at a density of 2 × 10^5^ cells/well. After being washed once with phosphate-buffered saline (PBS), the cells were fixed using 4% paraformaldehyde phosphate buffer solution (Nacalai Tesque, Inc., Kyoto, Japan) for 15 min at room temperature. After being washed with PBS containing 0.05% Tween 20 (PBST), the cells were permeabilized with 1% Triton X-100/PBS solution for 10 min and blocked with Blocking One Histo (Nacalai) for 30 min. Then, the cells were incubated with the primary antibodies for 1 h at room temperature in a humidified box. After rinsing the slides with PBST, the EnVision system (DAKO, Hamburg, Germany) was used to visualize antibody–antigen reactions according to the manufacturer’s procedure. The immunostained slides were examined under a microscope (Leica, Bensheim, Germany).

The primary antibodies were as follows: mouse monoclonal anti-cytokeratin 18 (CK18; Millipore); mouse monoclonal anti-cytokeratin 19 (CK19; Progen, Heidelberg, Germany); mouse monoclonal anti-α-smooth muscle actin (SMA; Progen); mouse monoclonal anti-macrophage scavenger receptor A (MSR-A:CD204) (KT022; TransGenic, Inc., Kumamoto, Japan); rabbit polyclonal anti-ionized calcium-binding adaptor molecule 1 (Iba1; Wako); and mouse monoclonal anti-CD172a (VMRD, Inc., Pullman, WA, USA). Since we have already confirmed that the KT022, Iba1, and CD172a antibodies react with antigens expressed by porcine primary macrophages (12, 13), these antibodies were used as markers of porcine macrophages.

### Phagocytic Assay Using Fluorescein Isothiocyanate (FITC)-Labeled Polystyrene Microbeads and pHrodo-Labeled *Escherichia coli* BioParticles

Fluorescein isothiocyanate-labeled polystyrene microbeads (diameter: 1.0 µm, #17154, Polysciences, Inc., Warrington, PA, USA) were diluted at 1:800 in growth medium, before being added to macrophages that had been seeded in plastic dishes (4 × 10^5^ cells/35-mm dish for primary PKM, and 1 × 10^6^ cells/60-mm dish for IPKM). After being incubated at 37°C, the cells were harvested with TrypLE Express (Thermo Fisher Scientific) at the time points indicated, rinsed with PBS three times to remove non-phagocytosed beads, and fixed with 3.7% formalin in PBS at room temperature for 15 min. After being washed with PBS, the cells were suspended in 0.5 ml of IsoFlow (Beckman Coulter, Fullerton, CA, USA) and then analyzed with a flow cytometer (Epics XL-MCL, Beckman Coulter) to investigate the phagocytosis of FITC-labeled microbeads.

Immortalized porcine kidney-derived macrophages (5 × 10^5^) were also cultured in 35-mm glass-bottomed dishes (Asahi Glass Co., Ltd.) containing growth medium. The next day, 20 µg/ml of pHrodo-labeled *E. coli* BioParticles (Thermo Fisher Scientific) were added, and the cells were subjected to time-lapse recording at 37°C for 4 h using an inverted fluorescence microscope (Olympus IX-81, Tokyo, Japan). The intensity of the fluorescence emitted by the pHrodo was quantified based on the captured photographs using the MetaMorph software version 7.6 (Molecular Devices, Downingtown, PA, USA).

### Cytokine Production

The macrophages were seeded in 60-mm plastic dishes at a density of 1 × 10^6^ cells/dish. The next day, the medium was replaced by growth medium containing lipopolysaccharide (LPS) (from *E. coli* serotype O127:B8, Sigma L3129) at doses of 0.1–1.0 µg/ml. After the cells had been incubated for 24 h at 37°C, the culture supernatant was collected, filtered with a membrane filter (pore size: 0.45 µm, Millipore Millex), and stored at −80°C until use. Aliquots of the samples were assayed using porcine cytokine enzyme-linked immunosorbent assay (ELISA) kits (R&D Systems, Minneapolis, MN, USA), according to the manufacturer’s instructions. The experiments were independently performed three times, and the cytokine concentrations of the culture supernatant are expressed as mean ± SEM values.

### Immunoblotting

The macrophages (3 × 10^5^ cells/well in a 24-well plate) were primed with 1 µg/ml LPS for 4 h, and then the medium was replaced with 250 µl HEPES-buffered salt solution (145 mM NaCl, 2.5 mM KCl, 1 mM MgCl_2_, 1.8 mM CaCl_2_, 20 mM HEPES, 10 mM glucose, and 0.01% bovine serum albumin; pH 7.4) containing nigericin (Sigma) or ATP (Sigma). After being incubated at 37°C for 30 min, the supernatants were collected, and the cells were lysed with 200 µl ice-cold lysis buffer. Maturation and secretion of interleukin (IL)-1β were evaluated using immunoblotting, as described previously (17). The target protein was revealed using ImmunoStar LD (Wako) and detected using a C-DiGit blot scanner (LI-COR, Inc., Lincoln, NE, USA).

### PCR Analyses

To discriminate between wild type and LDLR-KO pig-derived macrophages, PCR analyses were performed using KOD-FX (Toyobo, Tokyo, Japan), according to the manufacturer’s instructions. The cells (5 × 10^4^) were directly introduced into the PCR reaction. The following oligonucleotide primers were used: porcine LDLR: sense, 5′-GCAAGATAGGGGACTTTAGC-3′, and antisense, 5′-GCAAGATAGGGGACTTTAGC-3′; FP2: antisense, 5′-ACCAAATTAAGGGCCAGCTC-3′. The FP2 primer was designed based on a sequence within the targeted genomic region of LDLR-KO pigs (15). In addition, DNA fragments containing the targeted genomic region were amplified by PCR and cloned into the pBluescript cloning vector, and the vector plasmid was used as a positive control for the detection of the targeted locus. The PCR products were electrophoresed on agarose gel and visualized *via* GelGreen™ staining (Biotium, Inc., Hayward, CA, USA).

## Results and Discussion

### Immortalization of Primary PKM

Since isolated primary PKM that have become attached to plastic dishes cease to proliferate under culture conditions that do not include growth promoters (e.g., granulocyte-macrophage colony-stimulating factor or colony-stimulating factor 1), it is easy to determine whether a cell has acquired the ability to proliferate indefinitely. Based on the findings of a previous study (9), primary PKM were transfected with the pSV3-neo plasmid (ATCC Number: 37150) using the Lipofectamine (Thermo Fisher Scientific) or FuGENE HD (Promega) transfection reagent. However, we were unable to obtain an immortalized cell line that retained the characteristics of macrophages from these transfected cells. This was probably because the primary PKM were sensitive to the toxic effects of these transfection reagents.

Thus, we decided to use lentiviral vectors to transfer immortalizing genes into the primary PKM. Initially, the cells were infected with lentiviral particles carrying the gene for SV40LT. However, no immortalized cells were generated from the SV40LT-transfected PKM. Next, the cells were transfected with lentiviral particles carrying the SV40LT gene in combination with lentiviral particles carrying the pTERT gene. After several weeks of culturing, proliferative cells appeared in the plastic dishes and were routinely passaged. The expression of both SV40LT and pTERT mRNA was detected in these cells by Q-PCR analyses (data not shown), suggesting that the co-expression of SV40LT and pTERT was effective at immortalizing the primary PKM. Thus, we successfully produced an IPKM cell line.

### Immunocytochemical Characterization of the IPKM

**Figure 1:**
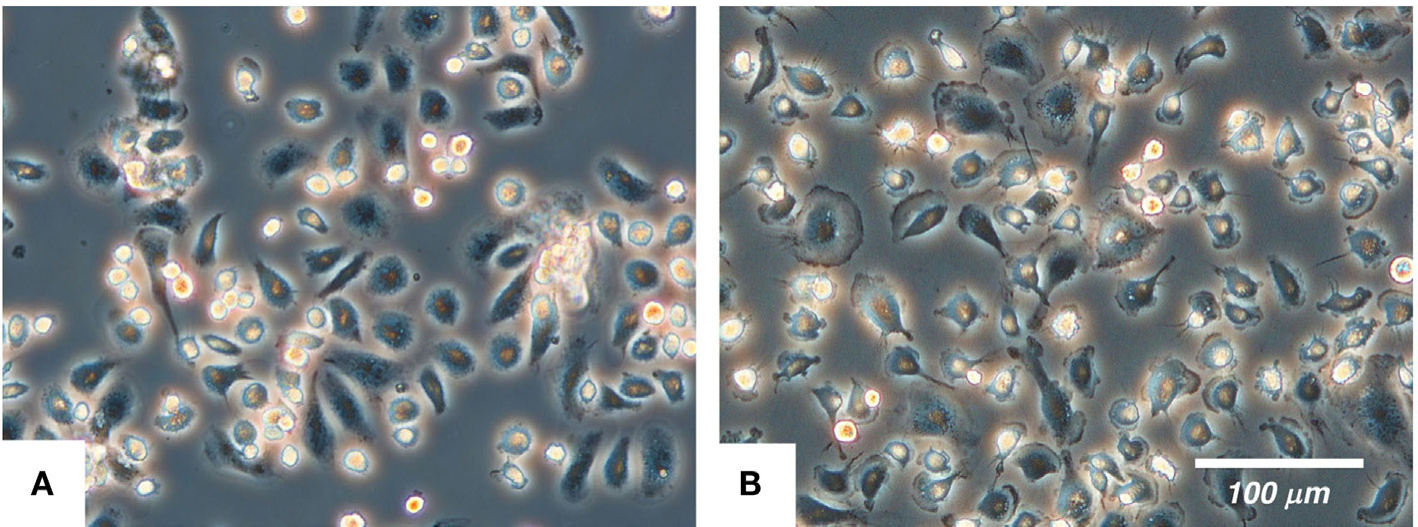
The morphology of the primary porcine kidney-derived macrophages **(A)** and immortalized porcine kidney-derived macrophages **(B)** in culture. The cells were attached to non-tissue culture grade plastic dishes and were examined under a phase-contrast microscope.

**Figure 2:**
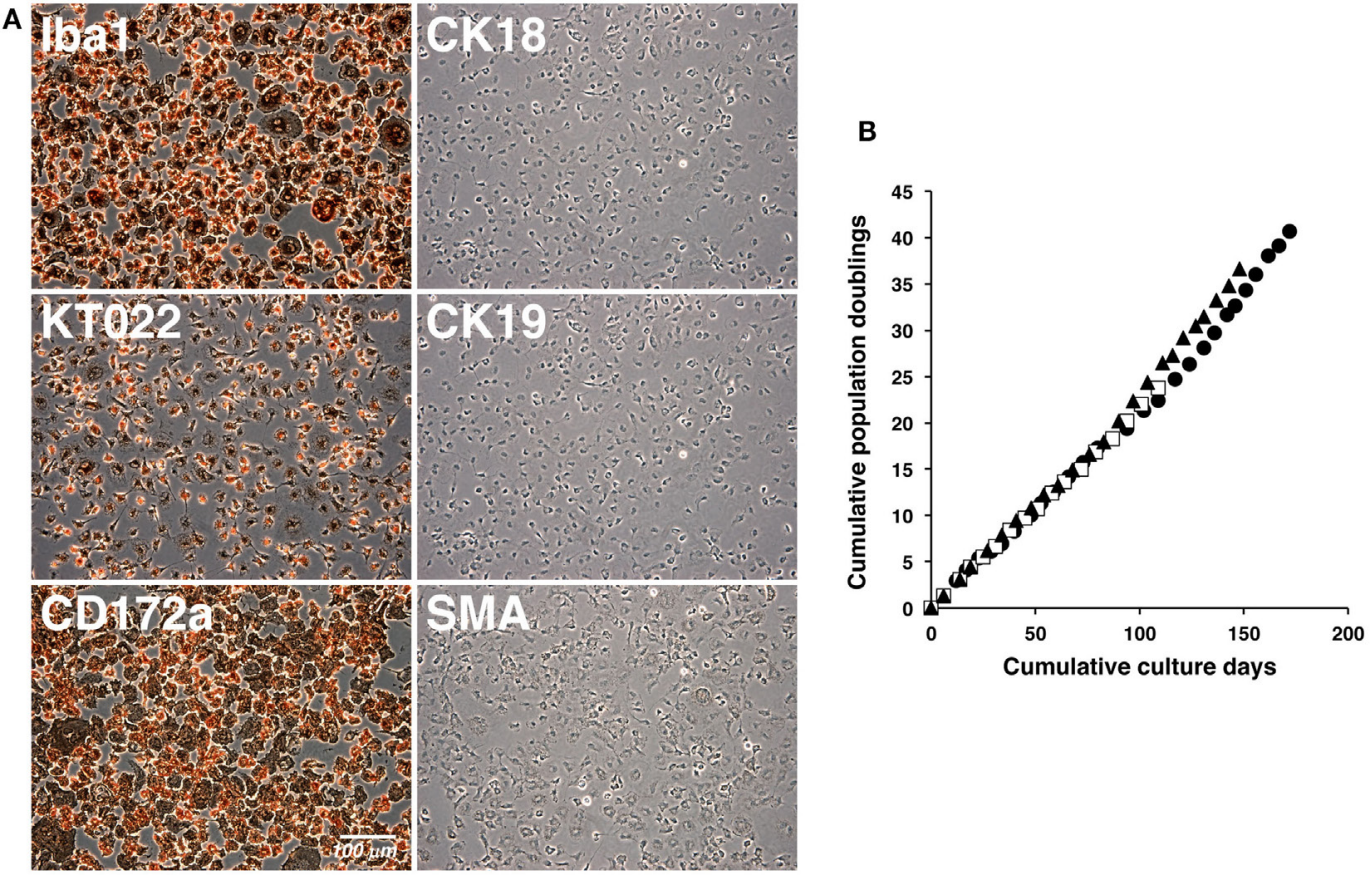
Immunocytochemical characterization and proliferation curves of the immortalized porcine kidney-derived macrophages (IPKM). IPKM were fixed and immunostained with specific antibodies against macrophage (Iba1, KT022, and CD172a), epithelial (CK18 and CK19), and mesenchymal (SMA) cell markers **(A)**. The cumulative population doublings of the IPKM were plotted against the duration of the culture period (in days) **(B)**. Three independent passage experiments gave similar proliferation curves (closed circles, closed triangles, and open squares) **(B)**.

The cultured IPKM exhibited a typical macrophage-like morphology with ruffled membranes and cell processes (Figure 1B); i.e., they resembled primary PKM (Figure 1A). Immunostaining demonstrated that the IPKM were positive for macrophage markers (Iba1, KT022, and CD172a), but negative for epithelial (CK18 and CK19) and mesenchymal (SMA) cell markers (Figure 2A), as was observed in the primary PKM (12). Regarding other cell surface markers of macrophages, although IPKM seem to express CD16 and major histocompatibility complex class II (Figure S1 in Supplementary Material), further experiments will be required to reveal the marker expression profiles of these cells. The IPKM proliferated at a doubling time of around 4 days and were stably passaged for >40 population doublings (Figure 2B).

### Phagocytic Activity and Cytokine Release of the IPKM

**Figure 3:**
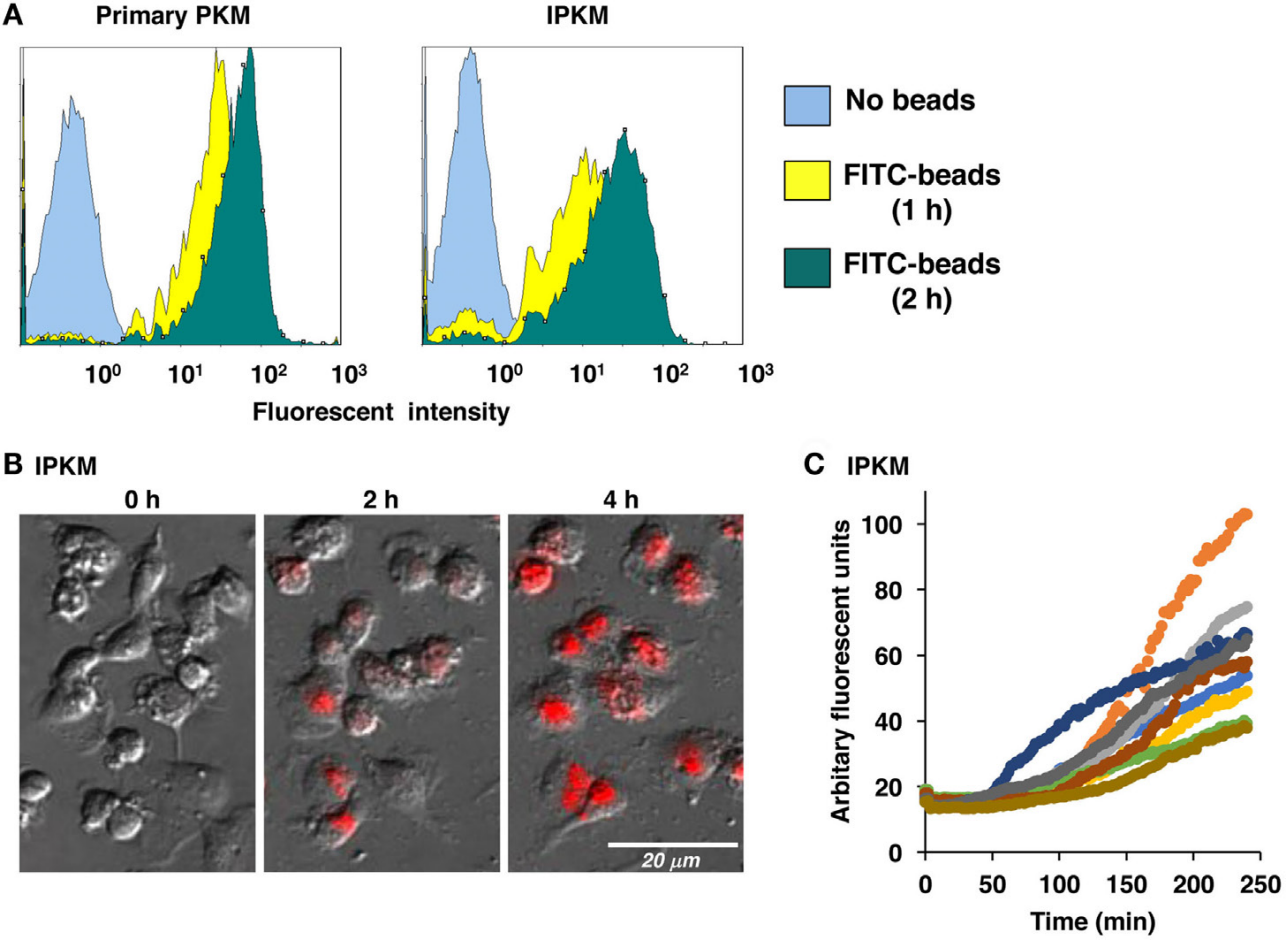
Phagocytosis of fluorescein isothiocyanate (FITC)-labeled microbeads and pHrodo-labeled *Escherichia coli* BioParticles by primary porcine kidney-derived macrophages (PKM) and immortalized porcine kidney-derived macrophages (IPKM). After primary PKM and IPKM had been incubated with FITC-labeled microbeads for 1 or 2 h, the number of fluorescence-positive cells was analyzed with a flow cytometer **(A)**. IPKM were also treated with pHrodo-labeled *E. coli* BioParticles, and changes in their fluorescence were detected after 2 or 4 h incubation using fluorescence microscopy **(B)**. The pHrodo-derived fluorescence intensity values obtained in each time-lapse recording of 10 cells are expressed in arbitrary units **(C)**.

Macrophages are professional phagocytes belonging to the innate immune system. The phagocytic activity of the IPKM was quantitatively evaluated using flow cytometry. After the addition of FITC-labeled beads to the IPKM culture, the proportion of fluorescence-positive IPKM increased to >95% within 2 h (Figure 3A). The levels of phagocytosis did not differ significantly between the primary PKM and IPKM (Figure 3A). To evaluate phagosomal maturation, IPKM were treated with *E. coli* BioParticles that had been covalently conjugated with pHrodo dye. The intensity of the fluorescence produced by this dye increases if the pH of its surroundings becomes more acidic (18, 19). Cells exhibiting pHrodo-derived fluorescence appeared after around 30 min incubation, and the number of these cells gradually increased in a time-dependent manner (Video S1 in Supplementary Material). pHrodo-labeled *E. coli* BioParticles that had been incorporated into IPKM displayed very bright fluorescence after 4 h incubation (Figures 3B,C), indicating that the phagosomes containing the BioParticles matured into phagolysosomes by fusing with lysosomes, which caused them to become more acidic. These results demonstrate that the IPKM exhibited substantial phagocytic activity.

**Figure 4:**
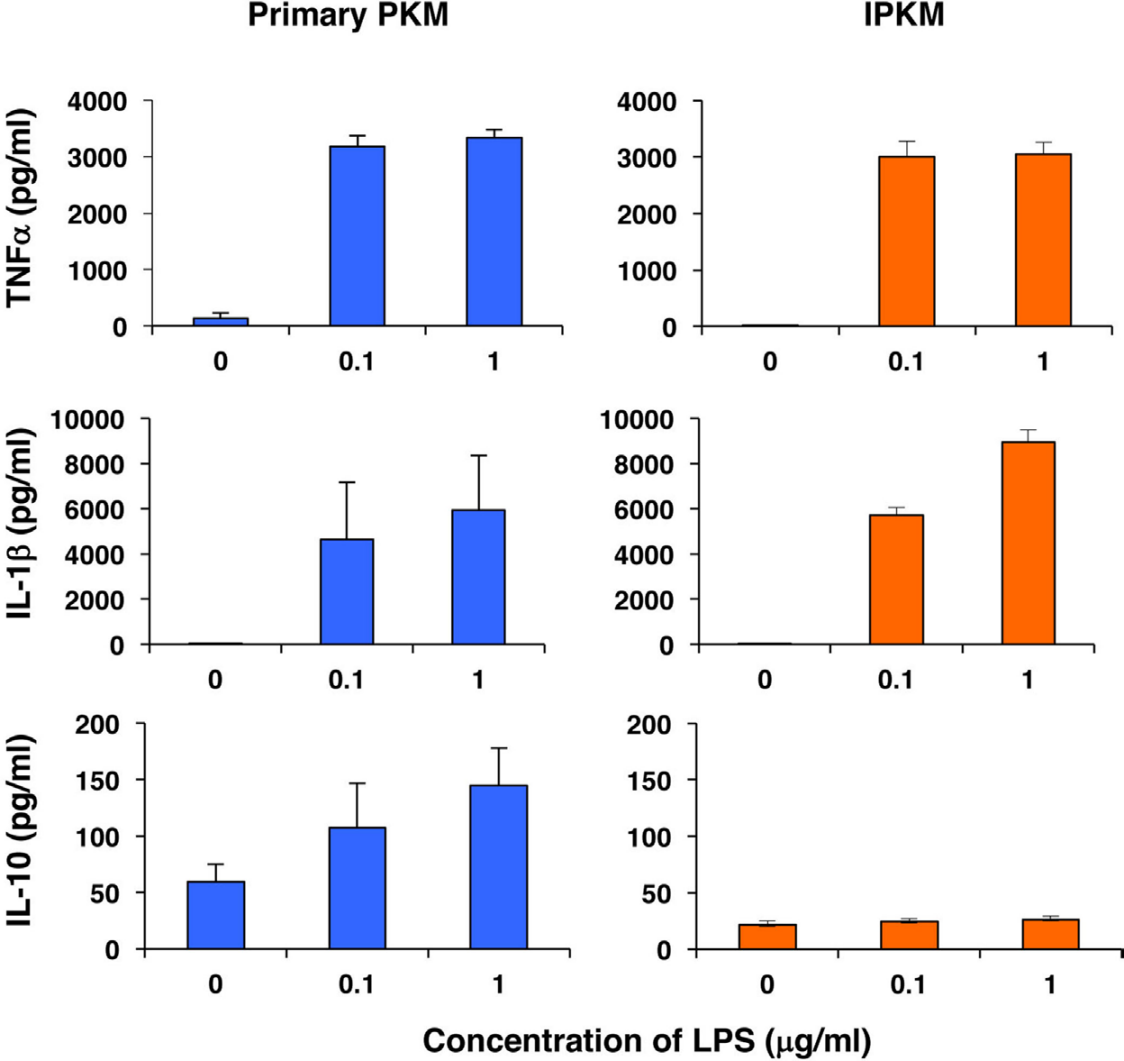
Secretion of inflammatory [tumor necrosis factor α (TNFα) and IL-1β] and anti-inflammatory (IL-10) cytokines from primary porcine kidney-derived macrophages (PKM) (left) and immortalized porcine kidney-derived macrophages (IPKM) (right) after stimulation with lipopolysaccharide (LPS) for 24 h. The cytokine concentrations of the culture supernatant were quantified using specific porcine enzyme-linked immunosorbent assay kits and are expressed as mean ± SEM values (*n* = 3).

Furthermore, we assessed the capacity of the IPKM to produce inflammatory and anti-inflammatory cytokines in response to stimulation with LPS. As was found for the primary PKM, the IPKM secreted substantial amounts of inflammatory cytokines, such as tumor necrosis factor α and IL-1β, after being stimulated with LPS for 24 h (Figure 4, upper and middle panels). In contrast, although the primary PKM secreted a substantial amount of the anti-inflammatory cytokine IL-10 upon LPS stimulation, the IPKM failed to secrete measurable amounts of IL-10 (Figure 4, lower panel). These findings suggest that the IPKM primarily displayed the pro-inflammatory M1 phenotype in response to bacterial endotoxins (20). Further studies using various stimuli are required to determine whether IPKM can exhibit the anti-inflammatory M2 phenotype.

### Inflammasome Activation in IPKM

**Figure 5:**
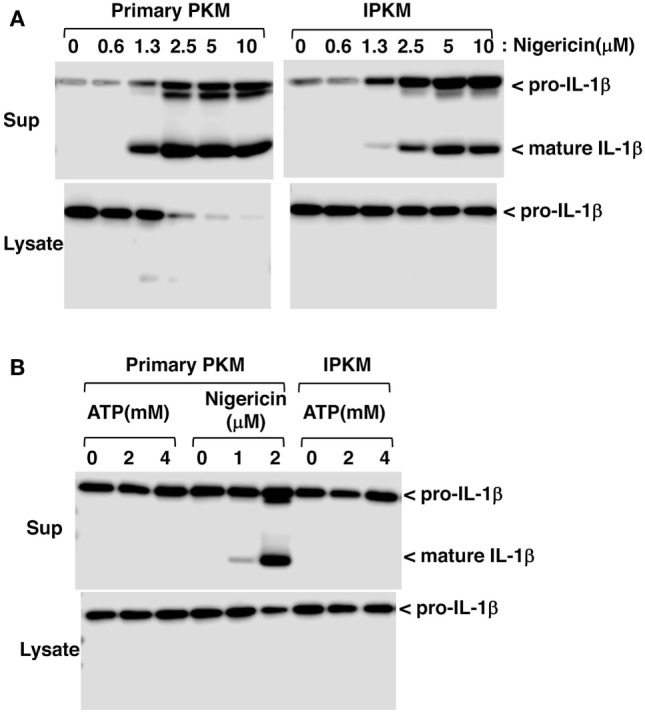
Production and secretion of mature IL-1β from lipopolysaccharide-primed primary porcine kidney-derived macrophages (PKM) and immortalized porcine kidney-derived macrophages (IPKM). Nigericin triggered the production and secretion of mature IL-1β in the IPKM in a dose-dependent manner **(A)**. The production/secretion of mature IL-1β was not detectable in the IPKM, even after their stimulation with millimolar concentrations of ATP **(B)**.

Inflammasomes are large intracellular multiprotein complexes that function during inflammatory immune responses (21). The activation of inflammasomes triggers caspase-1 activation followed by the maturation and secretion of IL-1β in macrophages. To verify the functional expression of the inflammasome system in IPKM, we assessed the effects of nigericin on the production and secretion of mature IL-1β in these cells. Nigericin is a potassium ionophore and is recognized as an activator of NLRP3 inflammasomes in humans and rodents (22). Nigericin triggered the production and secretion of mature IL-1β in the IPKM in a dose-dependent manner (Figure 5A). This suggests that IPKM are useful for studying the porcine NLRP3 inflammasome system.

Extracellular ATP is also known to be an activator of NLRP3 inflammasomes, and the P2X7 purinergic receptor (P2X7R), an ATP-gated cation channel, plays a critical role in NLRP3 inflammasome activation by ATP (23). In contrast to rodent macrophages, it has previously been demonstrated that the ATP/P2X7R pathway is impaired in primary PKM (12). Therefore, the effects of extracellular ATP on inflammasome activation were assessed in IPKM. As expected, extracellular ATP (in the millimolar range) failed to induce the production or secretion of mature IL-1β in IPKM, as was observed in the primary PKM (Figure 5B). This supports the notion that the IPKM originated from porcine macrophages and did not arise due to the contamination of the mouse macrophage cell line that was previously established in our laboratory (12, 17).

### Immortalization of Primary LDLR-KO Pig Blood-Derived Macrophages

**Figure 6:**
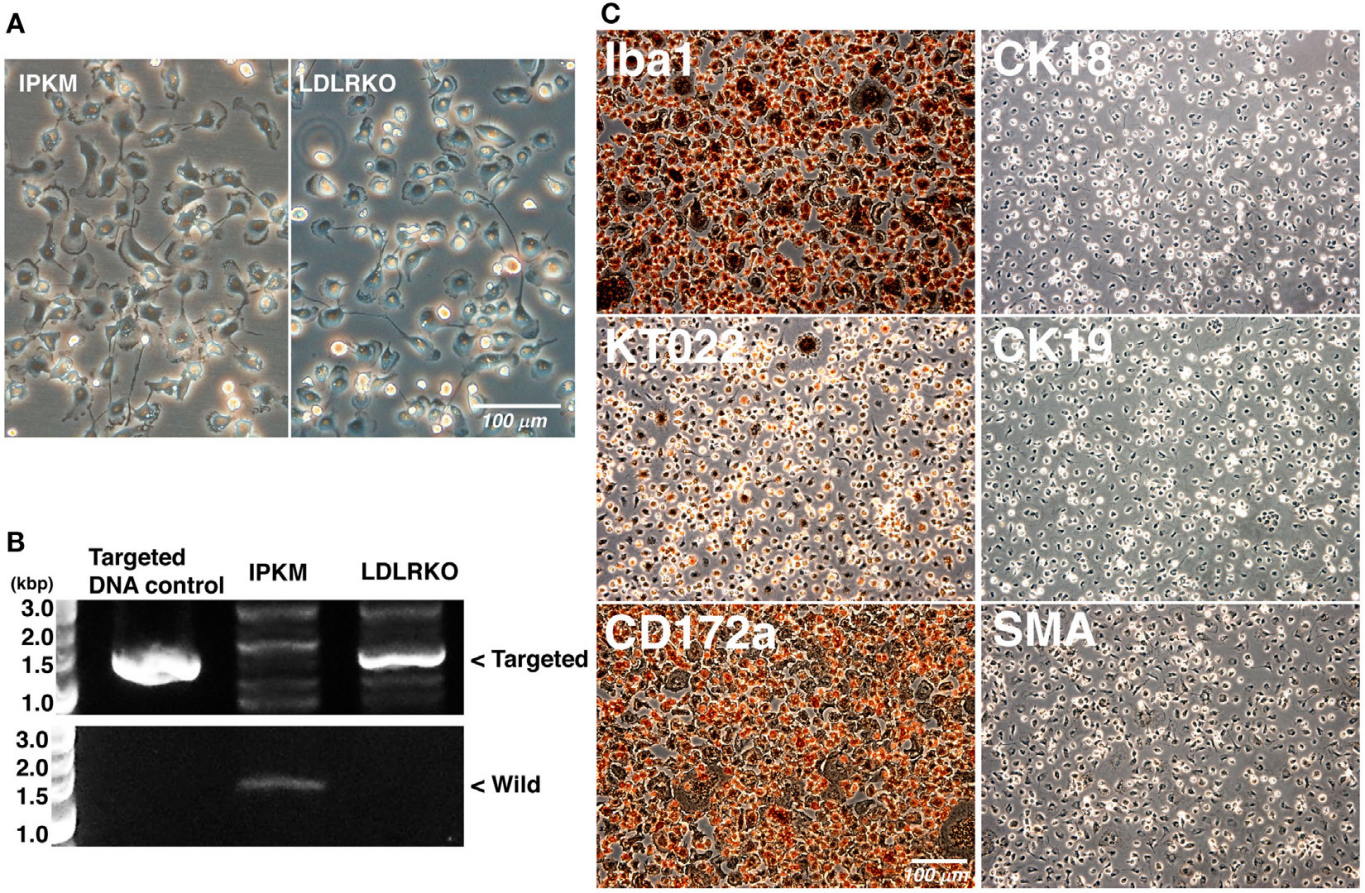
Characterization of immortalized macrophages derived from the peripheral blood of a low-density lipoprotein receptor knockout (LDLR-KO) pig. The morphology of the immortalized cells (LDLR-KO) closely resembled that of the immortalized porcine kidney-derived macrophages (IPKM); i.e., they exhibited ruffled membranes and cell processes in culture **(A)**. An amplified DNA fragment derived from the targeted genomic locus of the LDLR-KO pigs was detected in these cells, but not in the IPKM [**(B)**, upper panel]. An amplified DNA fragment derived from the wild-type LDLR genomic locus was only detected in the IPKM [**(B)**, lower panel]. The cells were also fixed and immunostained with specific antibodies against macrophage (Iba1, KT022, and CD172a), epithelial (CK18 and CK19), and mesenchymal (SMA) cell markers **(C)**.

To further verify the effects of the co-expression of SV40LT and pTERT on the immortalization of porcine macrophages, we attempted to immortalize LDLR-KO pig blood-derived macrophages. When LDLR-KO pig blood-derived macrophages were infected with lentiviral particles carrying the SV40LT gene in combination with lentiviral particles carrying the pTERT gene, immortalized cells were generated and routinely passaged. The morphology of the immortalized cells closely resembled those of the IPKM and primary macrophages; i.e., they have ruffled membranes and cell processes (Figure 6A). The presence of the targeted genomic locus in the immortalized cells was clearly shown by PCR (Figure 6B). As was found for the IPKM, immunostaining demonstrated that the immortalized cells were strongly positive for macrophage markers, but negative for other cell markers (Figure 6C). These findings suggest that inducing the co-expression of SV40LT and pTERT is an effective way of immortalizing porcine macrophages.

## Conclusion

This study describes a novel immortalized porcine macrophage cell line, IPKM, which was produced through the transduction of primary PKM with lentiviral vectors encoding SV40LT and pTERT. Using the same lentiviral transduction system, primary macrophages that were isolated from the peripheral blood of an LDLR-KO pig were successfully immortalized, suggesting that this protocol could be used to immortalize primary porcine macrophages. Since the IPKM retained the characteristics of primary macrophages, this cell line could be used to develop *in vitro* cellular models for studying genes associated with innate immunity in swine and porcine pathogen infection processes.

## Ethics Statement

Protocols for the use of animals were approved by the Animal Care Committee (#H28-P04) of the Institute of Agrobiological Sciences, National Agriculture and Food Research Organization (NARO). LDLR-KO pigs were conventionally reared in the Institute of Livestock and Grassland Science, NARO. Experiments using lentiviral vectors were approved by the Gene Recombination Experiment Safety Committee (#1036465) of the Institute of Agrobiological Sciences, NARO.

## Author Contributions

TT and HK performed the experiments and wrote the manuscript. SS, MN, DF, MT, HS, MS, and HU contributed reagents, materials, and/or analytical tools.

## Conflict of Interest Statement

The authors declare that the research was conducted in the absence of any commercial or financial relationships that could be construed as a potential conflict of interest.

## Supplementary Material

Video S1 Phagocytosis of pHrodo-labeled *Escherichia coli* BioParticles by immortalized porcine kidney-derived macrophages (IPKM). IPKM were treated with pHrodo-labeled *E. coli* BioParticles, and the changes in their fluorescence were monitored for 4 h using fluorescence microscopy and time-lapse recording.

Figure S1 Expression of CD16 and major histocompatibility complex class II (MHC-II) in immortalized porcine kidney-derived macrophages (IPKM).
