# Supplementary figures and images for: Immortalization and Characterization of Porcine Macrophages That Had Been Transduced with Lentiviral Vectors Encoding the SV40 Large T Antigen and Porcine Telomerase Reverse Transcriptase
Source: Front Vet Sci. 2017 Aug 21;4:132. doi: 10.3389/fvets.2017.00132 (PMC5566601; doi:10.3389/fvets.2017.00132)

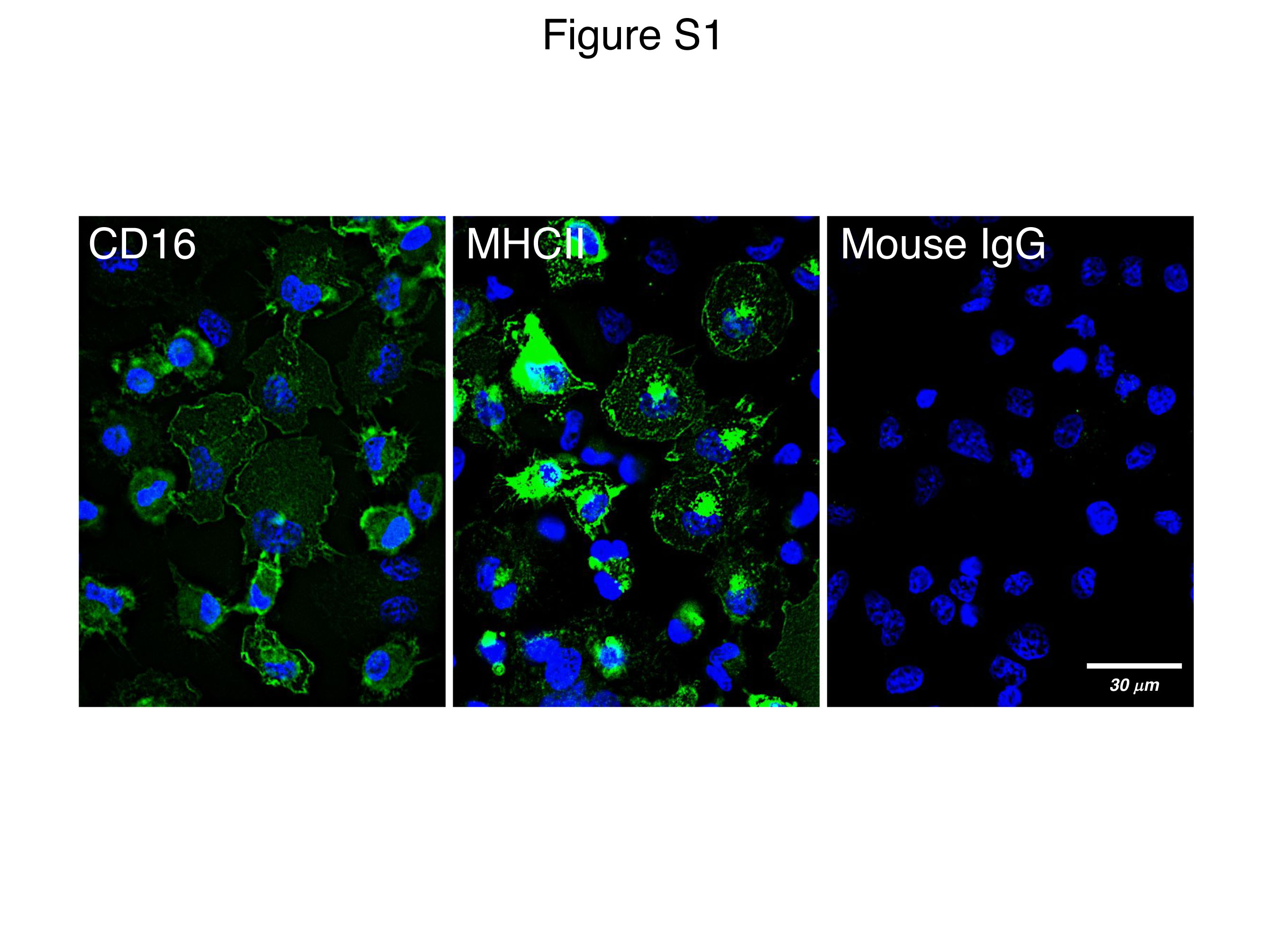

Supplement: Figure S1 — Expression of CD16 and major histocompatibility complex class II (MHC-II) in immortalized porcine kidney-derived macrophages (IPKM). [file image_1.jpeg]
