# Supplementary material for: Immortalization and Characterization of Porcine Macrophages That Had Been Transduced with Lentiviral Vectors Encoding the SV40 Large T Antigen and Porcine Telomerase Reverse Transcriptase
Source: Front Vet Sci. 2017 Aug 21;4:132. doi: 10.3389/fvets.2017.00132 (PMC5566601; doi:10.3389/fvets.2017.00132)
Supplement: Supplementary file 3 [file data_sheet_1.docx]

Supplementary Material:

Immortalization and characterization of porcine macrophages that had been transduced with lentiviral vectors encoding the SV40 large T antigen and porcine telomerase reverse transcriptase

Takato Takenouchi*, Hiroshi Kitani, Shunichi Suzuki, Michiko Nakai, Dai-ichiro Fuchimoto, Mitsutoshi Tsukimoto, Hiroki Shinkai, Mitsuru Sato and Hirohide Uenishi

* Correspondence:

Takato Takenouchi, Ph.D.

ttakenou@affrc.go.jp

**Supplementary Figure S1: Expression of CD16 and MHC-II in IPKM**

IPKM were seeded in 8-well chamber slides (Asahi Glass Co., Ltd.) at a density of 2×10^5^ cells/well. The cells were fixed using 4% paraformaldehyde phosphate buffer solution (Nacalai) for 15 min, and then blocked with Blocking One Histo (Nacalai) for 30 min. Next, the cells were incubated with FITC-labeled mouse monoclonal anti-pig CD16 antibody (Bio-Rad) (1:200) for 1 h. The cells were also incubated with mouse monoclonal anti-swine MHC-II antibody (Kingfisher Biotech) (1:400) and control mouse IgG (Sigma) (1:400) for 1 h, before being incubated with Alexa Fluor 488 goat anti-mouse IgG antibody (1:400) for 1 h. The immunostained cells were mounted using ProLong Gold antifade reagent with DAPI (blue) (Life Technologies), and observed using an inverted fluorescence microscope (Olympus IX-81). A z-stack comprising 50 images was taken at 0.2-μm intervals using a CCD camera (Retiga-SRV, Q-imaging Co., Surrey, BC, Canada) controlled by the MetaMorph software (Molecular Devices, Downingtown, PA). The z-stacks of fluorescence photographs were deconvoluted using a 3D-blind deconvolution algorithm (AutoQuant X software, Media Cybernetics, Bethesda, MD).

**Supplementary Video S1.** **Phagocytosis of pHrodo-labeled *E. coli* BioParticles by IPKM**

IPKM were treated with pHrodo-labeled *E. coli* BioParticles, and the changes in their fluorescence were monitored for 4 h using fluorescence microscopy and time-lapse recording.
